# Energy Modeling of IoT Mobile Terminals on WiFi Environmental Impacts †

**DOI:** 10.3390/s18061728

**Published:** 2018-05-28

**Authors:** Yuxia Sun, Junxian Chen, Yong Tang, Yanjia Chen

**Affiliations:** 1 Department of Computer Science, Jinan University, Guangzhou 510630, China; junxianchen001@gmail.com (J.C.); yanjiachen0505@gmail.com (Y.C.); 2 College of Computing, South China Normal University, Guangzhou 510630, China; ytang@scnu.edu.cn

**Keywords:** WiFi environment, phone energy consumption, signal strength, packet type, packet amount, modeling method

## Abstract

With the popularity of various IoT mobile terminals such as mobile phones and sensors, the energy problems of IoT mobile terminals have attracted increasingly more attention. In this paper, we explore the impacts of some important factors of WiFi environments on the energy consumption of mobile phones, which are typical IoT end devices. The factors involve the WiFi signal strength under good signal conditions, the type and the amount of protocol packets that are initiated by WiFi APs (Access Points) to maintain basic network communication with the phones. Controlled experiments are conducted to quantitatively study the phone energy impacts by the above WiFi environmental factors. To describe such impacts, we construct a time-based signal strength-aware energy model and packet type/amount-aware energy models. The models constructed in the paper corroborate the following user experience on phone energy consumption: (1) a phone’s energy is drawn faster under higher WiFi signal strengths than under lower ones even in normal signal conditions; (2) phones consume energy faster in a public WiFi network than in a private one even in the basic phone state. The energy modeling methods proposed in the paper enable ordinary developers to analyze phone energy draw conveniently by utilizing inexpensive power meters as measurement tools. The modeling methods are general and are able to be used for phones of any type and any platform.

## 1. Introduction

With the incredible popularity of IoT mobile devices all over the world, the energy consumption problems of such IoT terminals as mobile phones and sensors have gained growing attention. The constrained battery capacity of mobile phones or mobile sensors is a pain that users have to face while enjoying various energy-consuming applications. Thus, it is significantly important to understand and then optimize the energy consumption of such mobile devices. In this paper, we investigate the modeling problem of the energy consumption of typical IoT mobile terminals, namely mobile phones.

WiFi connection, as one prime way for mobile phone users to access the Internet, is a major source of mobile device energy consumption [1,2,3]. For instance, many mobile phone users experienced that, with the WiFi switched on, a phone’s energy drains at various rates under different WiFi environments, even if the phone keeps the same application state (e.g., running no applications) and maintains the same hardware settings in all the environments. As reported by prior work, WiFi environments have notable impacts on mobile phones’ energy draw [4,5,6]. In-depth studies have been conducted on how poor WiFi signal conditions obviously inflate the energy drain of mobile phones [7,8,9].

Most existing work on phone energy consumption in WiFi networks aims to reveal the direct cause of the phone energy consumption due to WiFi connection. Thus, the work primarily focuses on the energy consumption of the phones’ WiFi interface cards and analyzes the packet-sending-related parameters on the phones. Different from such work, our research aims to explain the following user experience on phone energy consumption in WiFi networks: (1) phones consume energy faster under higher WiFi signal strengths than under lower ones even when the WiFi signals are not weak; (2) phones consume energy faster in a public WiFi network than in a private one even in the basic phone state. We will create energy models to help users who are always non-network specialists to understand the above user experience. For this reason, our research scope is as follows:

1 We concentrate on two WiFi factors, namely the WiFi signal strength and the WiFi network type: public or private network;
2 We study the overall energy consumption by the hardware components of the display, CPU, memory, as well as the WiFi components on the phone.

To study the impact of WiFi signal strength on phone energy consumption, we conduct a group of experiments, detailed in Section 4, under normal WiFi signals whose RSSIs are over −50 dBm [8], rather than weak WiFi signals. We demonstrate how to construct a time-based energy model with WiFi signal strength as the parameter.

A phone user’s WiFi connection quality can be degraded in a public WiFi network compared to in a private one, which might due to the shared channel by many users or the phone’s mobility. As a result, re-connections to the public WiFi network will lead to unwanted energy draw on the phone. However, even when the WiFi connection quality is good and stable, phone energy drain in a public network can still be non-neglectably more than that in a private network. An important reason for this is that some data packets that can only be sent by public network APs (Access Points), rather than by private ones, and frequently wake the phone’s WiFi module, even if there is no related request made by phone applications, which potentially leads to much energy drain.

In this paper, we empirically investigate the impacts of WiFi factors on energy consumption of smart phones in the basic state: in a state where the phone runs no applications and keeps the WiFi switched on, keeps Bluetooth/GSM/3G radios disabled and camera/microphone/speakers off. When a phone is in its basic state, the energy consumption interference from various applications and non-WiFi radios can be excluded. To exclude the interference from other WiFi hotspots or devices, the experiments in this paper are performed with only one WiFi AP during the night hours, when interference was minimum. In our experiments, we measure the overall energy consumption of the mobile phone in the basic state. The energy draw is due to not only the WiFi NIC, but also other phone components including the CPU, memory and screen.

To study the impact of WiFi network type (namely public WiFi network or private one) on phone energy consumption, we perform another group of experiments, detailed in Section 5. We feature a public or private WiFi network environment using the packets sent by a WiFi AP to the phone, based on the following observations: to initiate a phone’ WiFi network connection and packet switching so as to keep the phone in the basic state, an AP in a private network primarily sends DHCP (Dynamic Host Configuration Protocol) packets and ARP (Address Resolution Protocol) to the phone, while an AP in a public network sends many DHCP packets, ICMP (Internet Control Message Protocol) packets and IGMP (Internet Group Management Protocol) packets. Here, DHCP packets sent by both networks are of the UDP (User Datagram Protocol) packet-type; ARP packets only available in private networks are encapsulated in TCP (Transmission Control Protocol) packets; while ICMP packets and IGMP packets are only sent by public network APs. Thus, in our experiments, we utilize the four network protocol packets (namely UDP packets, TCP packets, ICMP packets and IGMP packets) sent by WiFi APs to denote the two types of WiFi network environments. In other words, in our experiment, the WiFi AP sending ICMP packets, IGMP packets and UDP packets denotes a public WiFi network, while the WiFi AP sending TCP packets and UDP packets represents a private WiFi network. We focus on the packets that are initiated by APs to maintain basic communications, as detailed in Section 5.

By performing experiments, we explore the following research questions:

Q1: How does WiFi signal strength impact the energy consumption of a smart phone?
Q2: What energy model can be constructed to indicate the impact mentioned in Q1?
Q3: How do protocol packets initiated by WiFi APs impact the energy consumption of a smart phone?
Q4: What energy model can be constructed to indicate the impact mentioned in Q3?

Overall, as the wireless network is being widely used around the world, it is important to study such user-concerned WiFi environmental factors as signal strength, public or private network type on the energy consumption of smart phones [10] and to propose energy models to depict the impacts. It is noteworthy that although the model created in this paper is dependent on the types of the phones’ hardware components (i.e., WiFi, CPU, memory and screen components), the method to create the models could be applied to any phones. Our modeling methods only require an inexpensive power meter as the measurement tool, and the model parameters are easily available, thus enabling ordinary developers to analyze phone energy draw conveniently.

The rest of the paper is organized as follows. We discuss related work in Section 2. Section 3 details our method of measuring the phone energy consumption. Section 4 elaborates how WiFi signal strengths impact phone energy draw with a group of experiments and proposes a time-based energy model. Section 5 explains how packet type and amount impact phone energy drain by another group of experiments, together with energy models. Section 6 discusses the models and the results observed in the previous experiments. We conclude our work in the last section.

## 2. Related Work

### 2.1. Phone Energy Models of WiFi Components

(1) Phone energy models related to WiFi signal strength: The impact of WiFi signal strength on phone energy consumption has been studied in some previous work, but few energy models have been created based on WiFi signal strengths. For examples, as one of the early works, Gupta et al. [7] performed the measurement and analysis work on the phone energy draw related to WiFi signal strength and dynamic power control. Ding et al. [8] proposed a signal strength-aware model by systematically breaking down the impact of poor WiFi signal strength on phone energy drain. Sun et al. [9] were concerned with the phones’ energy consumption in active power states and proposed energy models based on the application layer throughput. They claimed that signal strength alone cannot always capture the dynamics of the wireless channel.

In this paper, we create an energy model of the entire phone in the basic state based on WiFi signal strength. We focus on the phone energy under good WiFi signals whose RSSIs are above −50 dBm and construct a signal strength-aware energy model based on time. The authors of [7,9] analyzed the impact of WiFi signal strength on phone energy consumption, but they did not create energy models on WiFi signal strength. The authors of [8] proposed a signal strength-aware model. However, different from our work, they focused on energy drawn by WiFi NICs under poor WiFi signals and modeled the energy without the variable of time.

(2) Phone energy models related to WiFi packets: Packet-driven phone energy models in WiFi networks have been reported in some prior work. For instance, Sun et al. [9] studied the properties of active power consumption models based on packet sizes and transport layer protocols in WiFi networks. Zhang et al. [11] presented an automated power model construction technique by using built-in battery voltage sensors and knowledge of battery discharge behavior. They created power models of WiFi components in different power states with the following variables related to TCP packets and UDP packets: number of packets transmitted and received per second, uplink channel rate and data rate. Khan et al. [12] developed energy models for 802.11n wireless cards to drive the design of an energy-aware rate adaptation scheme. They used UDP packets and constructed energy models for transmitting or receiving a frame based on the total amount of time required to successfully deliver a frame to the receiver. Xu et al. [13] utilized battery voltage dynamics to measure phone energy consumption and create energy models for phone components. They proposed a simple WiFi model based on incoming and outgoing data through the WiFi NIC. Xiao et al. [14] presented energy models for WiFi data transmission with traffic burstiness, network performance metrics and parameters of the power saving mechanisms in use. Their models are applicable to both TCP and UDP transmission.

In this paper, we also created phone energy models based on WiFi data packets. We concentrated on the packets initiated by the AP, including ICMP packets and IGMP packets, as well as UDP packets and TCP packets. Different from our work, the authors of [9,11,14] modeled the phone energy based on TCP packets and UDP packets, while the authors in [12,13,15] used only UDP packets, varying the packet size and amount.

(3) Other phone energy models of WiFi components: For WiFi components of smartphones, some researchers have created energy models that correlate energy consumption to different factors from WiFi signal strengths and packets. For examples, Pathak et al. [16] proposed a power model based on system-calls that indicated the power actions of hardware components including WiFi components and used a Finite State Machine (FSM) to capture the power behavior of system calls. Balasubramanian et al. [17] empirically studied energy consumption of WiFi and other radio components of phones and formulated an energy model for WiFi based on the transfer energy cost and the maintenance cost of WiFi.

In this paper, we create phone energy models by correlating energy consumption with WiFi signal strengths or packets. Unlike our work, some prior work correlated energy consumption with other factors, such as system-calls initiated by the WiFi NIC [16], the transfer energy cost and the maintenance cost of WiFi [17], and so on.

### 2.2. Energy Consumption of Phone Components

Previous work has been done to measure and model the energy consumed by various phone components such as the CPU [18,19,20,21,22], memory [18,23], WiFi NIC [18,19,20,21,24], display screen [24], GPS sensor [2,11], Bluetooth module [25,26,27], cellular module [25], and so on.

In this paper, we measure and model the energy of an entire phone in the basic state in WiFi networks. The energy measured includes that consumed by the components of the CPU, memory, WiFi NIC and screen. Because a phone in the basic state runs no applications, keeps Bluetooth/GSM/3G radios disabled and camera/microphone/speakers off, the energy consumed by the other components (e.g., sensors, cameras, Bluetooth modules and cellular modules) is zero and not involved in our model. Compared to our work, the work mentioned in Section 2.2 does not aim to model the energy of the phone in the basic state, and the models are for different phone components [2,11,25,26,27] or for individual components [18,19,20,21,22,23,24], rather than the entire phone.

### 2.3. Phone Energy Saving in WiFi Networks

There were some prior works that aimed to reduce the phone energy consumption caused by WiFi networks. For example, Zhang et al. [28] put forward a system-level power management method to improve a phone’s standby time, by predicting Actionable Silent Periods (ASPs) of the WiFi interface and shutting the interface down during these ASPs. Xia et al. [29] proposed an A-GPS-assisted scheme to reduce the numbers of unnecessary WiFi scans in the non-connected state of a phone, by using the phone’s location information to find the nearest WiFi network AP. Choi et al. [30] also presented an energy-efficient WiFi scan system for localization, by optimizing the dwell time of beacon-listening to obtain the minimum number of scanned APs.

The energy model created in this paper can help app developers and mobile users analyze the phone energy draw in normal WiFi environments, thus enabling them to take appropriate measures to save phone energy.

## 3. Measurement of Energy Consumption

**Figure 1:**
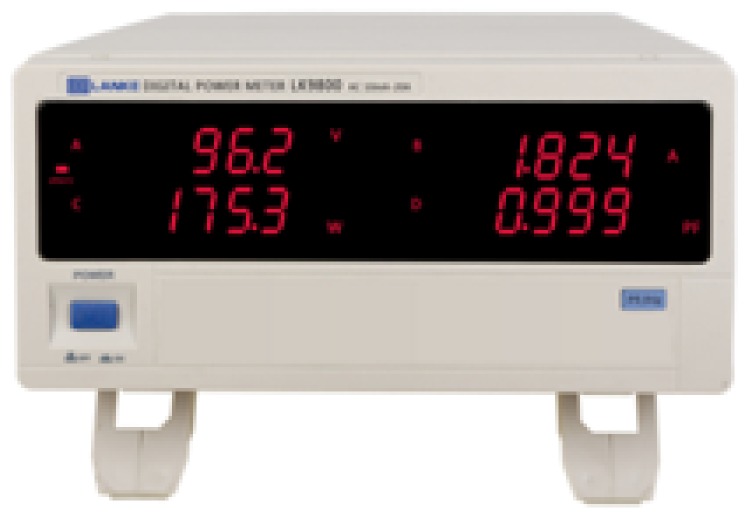
Power meter.

**Figure 2:**
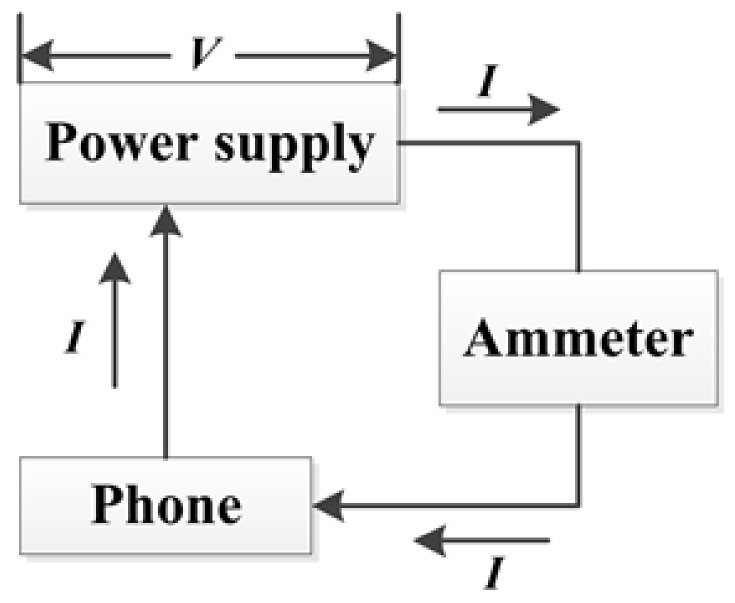
Power measurement.

Figure 1 shows the power meter used in our experiment, which consists of a power supply with adjustable stabilized voltage and a current meter. The sampling rate of the power meter shown in Figure 2 is current samples per second. Its resolution is 10 mV/1 mA, which enables sample collection with a very fine granularity, 10 mW, as required in [31].

To measure the overall system power of a smart phone, we unload the battery of the phone and connect the phone to the power meter. Figure 2 illustrates the connection. During all measurements, the voltage value *V* is adjusted to a constant. By reading the current value *I*, we can get the power drain value *P*, i.e., V*I, of the phone.

To measure the overall energy consumption of a smart phone during any *t* seconds, we collect all the current samplings using the above power meter. Because the sampling rate is two samples per second, the sampling duration *t* consists of 2t sampling periods, and each period is 0.5 s. The overall energy consumption of the smart phone within *t* seconds is calculated as follows:

(1) $$E\left(t\right)=\sum_{i=1}^{2t}VI_{i}*0.5=0.5V\sum_{i=1}^{2t}I_{i}$$

## 4. Impact of WiFi Signal Strength

In this section, we conduct experiments to investigate the impact of WiFi signal strength on phone energy, i.e., to answer research questions Q1 and Q2. The signal strength is measured as the RSSI (Received Signal Strength Indicator) level.

### 4.1. Energy Measurement

In this experiment, the smart phone under measurement is a Samsung Galaxy GT-S7898 phone running on Android 4.1.2. During the measurement, we keep the phone in the basic state by turning off all the applications and keep the WiFi switched on. We use an AP set on a PC to transmit WiFi signals. In a WiFi network, a phone’s received signal strength is widely used to infer the phone-AP distance, and weak WiFi signal strength always indicates long distance from the AP [32]. The existing models [32,33,34,35] indicate that the WiFi signal strength reduces with the increase of the phone-AP distance. Thus, to control the WiFi signal strength, we vary the distance between the AP and the smart phone. The RSSI level is measured using a software tool named Wirelessmon [36] running on a laptop computer. By placing the laptop at a spot to measure the phone energy, we can collect the corresponding WiFi signal strength at that spot.

We adjust the phone-AP distance, ranging from 0.5 m to 4 m with an interval of 0.5, by placing the phone at eight spots. At each spot, we monitor the total energy consumption of the phone for a period of time (for instance, 30 s). Table 1 lists the results acquired at the end of 30 s. The first column shows the eight phone-AP distances measured in meters. The second column denotes the signal strengths, measured as the RSSI level, at each spot. The last column represents the total energy consumption (Joule) of the phone within 30 s for each RSSI level. We repeat the experiments 10 times. In the 10 experimental runs, for one distance value, we got the same RSSI value (as Column 2 of Table 1 shows) and 10 different energy consumption values. We compute the mean value and the square error and show them in the last column of Table 1.

**Figure 3:**
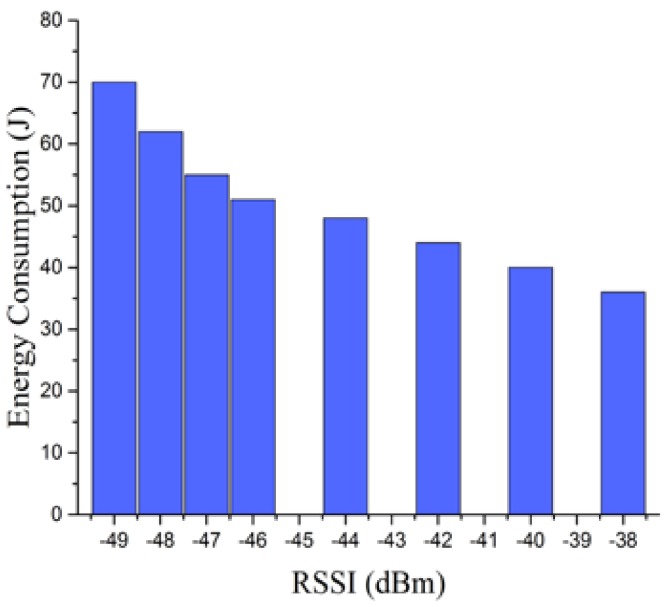
Energy consumption in 30 s vs. RSSI.

To visualize the above results, we use Figure 3 to demonstrate the total energy consumption of the phone within 30 s under four RSSI levels. We make the following observation from the graph in Figure 3: with the signal strength increased (e.g., from −49 dBm to −38 dBm), that is with the phone-AP distance decreased (e.g., from 4 m to 0.5 m), the energy consumption of the phone can be reduced obviously (e.g., from 70 J to almost half, namely 36 J).

At each measurement spot, we measure the energy consumption of the smart phone during 30 s by utilizing the methodology described in Section 3. The stabilized voltage V of the power meter is set to a constant of 4.2 V. With the power meter, we can log 60 current values within 30 s. Then, we can calculate the corresponding energy values along time according to (1).

**Figure 4:**
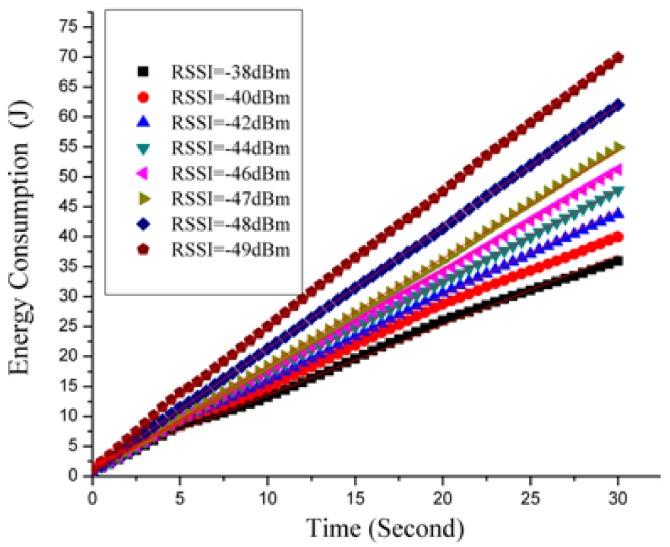
Energy consumed along time.

Figure 4 plots the phone energy consumption (Joule) sampled along 30 s under the four RSSI levels mentioned above. Eight types of scatterplots are made by different point types and point colors. For example, under the RSSI level of −40 dBm, the scatterplot consists of 60 black and square sampling points. We can make the following observation from the graph in Figure 4:

- Under a given signal strength (i.e., RSSI level), the total energy draw of the phone increases with time in a near-linear trend.
- During each sampling period, under higher signal strength (i.e., higher RSSI value), the total energy draw of the phone is lower.

### 4.2. Energy Model

**Figure 5:**
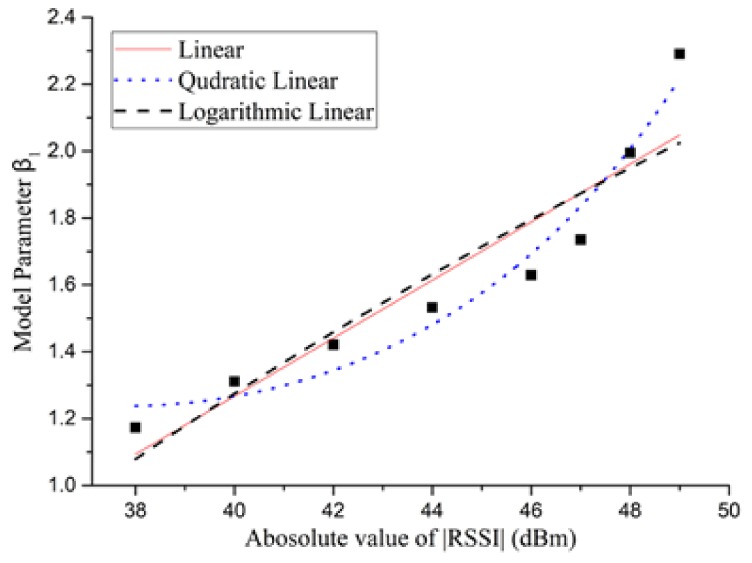
|RSSI| vs. energy model parameter $\beta_{1}$.

**Figure 6:**
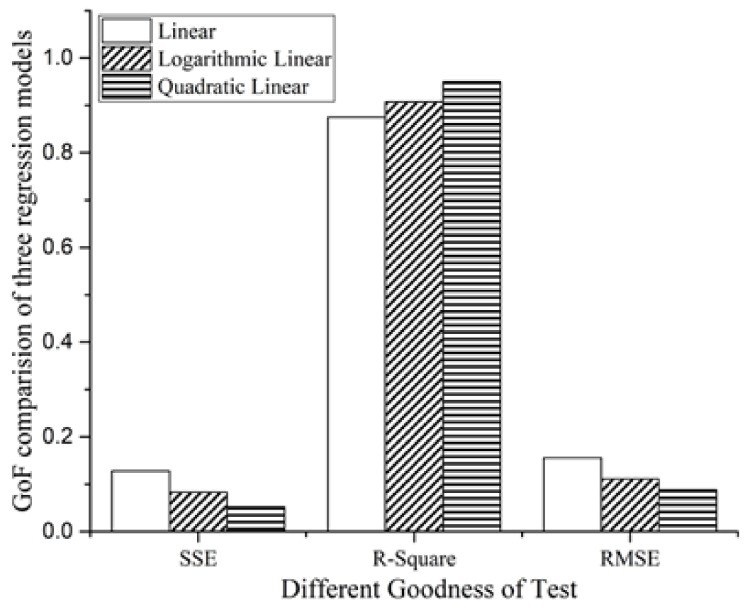
Different goodness of fit tests for the three models.

This subsection aims at introducing a simple measurement-time-based model for estimating the energy consumption of smart phones as a function of the WiFi signal strength. We use three curve fitting approaches compared with each other to develop the best model that is able to match the energy consumption with RSSI and the time values exhibited in Figure 4 well, and the detailed steps are as follows:

(1) Create linear regression models of energy consumption vs. time under given RSSI levels: For each given RSSI level, Figure 4 illustrates a curve fitting process of the energy consumption with time, and the corresponding linear regression equation is in the following form:
  (2) $$E\left(t\right)=\beta_{1}t+\beta_{0}$$
  In Equation (2), E(t) is the total energy consumption of the phone within the time period *t*; $\beta_{0}$ and $\beta_{1}$ are two model parameters.
  (a) Determine the $\beta_{0}$ value: Under the RSSI level of −38 dBm, we set up a linear regression model for energy draw vs. time, shown in Column 2, Row 2 in Table 2, by utilizing the statistical values of the 60 black points plotted in Figure 4. As the model demonstrates, the parameter $\beta_{0}$ is fitted to 1.76.
  (b) Determine the $\beta_{1}$ value: For the other RSSI levels of −40 –−49 dBm, we use the energy values vs. time plotted in Figure 4, together with the $\beta_{0}$ value of 1.76, to create seven linear regression models, as shown from Row 3–Row 9 of Column 2 in Table 2.
  (c) Validate models: To evaluate the model predicted data deviation from the experimental data, we perform three GoF (Goodness of Fit) tests, namely SSE (Sum of Squared Errors), R-square value and RMSE (Root Mean Squared Error). The SSE and RMSE tests are based on affinity to zero, while the R-square value should approximate to one. Suppose that $y_{i},\;{\hat{y}}_{l}$ and $\bar{y}\;\left(0\leq i\leq 60\right)$ represent experimental data, model predicted data and average experimental data, respectively, and *v* denotes the difference between the number of experimental data and the number of adjustable parameters, then the three test methods are formulated as follows [37]:
    (3) $$SSE=\sum_{i=1}^{n}{\left(y_{i}-{\hat{y}}_{l}\right)}^{2}$$
    (4) $$R-square=1-\frac{SSE}{\sum_{i=1}^{n}{\left(y_{i}-\bar{y}\right)}^{2}}$$
    (5) $$RMSE=\sqrt{\frac{SSE}{v}}$$
    Table 2 shows the three GoF test results from Column 3–5: R-square values are close to one, and the results from the three tests are consistent with each other. It is observed that our models are able to predict experimental data very well using the error analysis methods SSE, R-square value and RMSE. Therefore, under a given RSSI level, the following model of energy consumption with time is reliable:
    (6) $$E\left(t\right)=\beta_{1}t+1.76$$
    In (6), the value of $\beta_{1}$ depends on the value of the RSSI level, as shown in Column 1 and 2 in Table 2.
(2) Create the regression model of |RSSI| vs. $\beta_{1}$: Figure 5 illustrates three curve fitting methods of the absolute value, |RSSI|, with the parameter $\beta_{1}$, where the eight |RSSI| values (namely, 38, 40, 42, 44, 46, 47, 48 and 49) are derived from Column 2 in Table 1 and the eight $\beta_{1}$ values (from 1.173 to 2.291) are derived from Column 2 in Table 2. Accordingly, we set up the linear regression, quadratic linear regression and logarithmic linear regression models for $\beta_{1}$ with |RSSI|, and Figure 6 compares the GoF tests of the three models to select the best one to explain the relationship between the |RSSI| and $\beta_{1}$ suitably. From Figure 6, we can see that the quadratic linear regression model has the largest value of R-square, and the SSE and RMSE values are smaller than the other two methods. We can get the quadratic relation as follows:
  (7) $$\beta_{1}={0.009|RSSI|}^{2}-0.7\left|RSSI\right|+14.87$$
(3) Create the target energy model: By replacing $\beta_{1}$ in (6) with the right part of (7), we can get the energy model with time and RSSI levels as follows:
  (8) $$E\left(RSSI,t\right)={\left(0.009|RSSI\right|}^{2}-0.7|RSSI\left|+14.87\right)t+1.76$$
  The energy model (8) is a function of WiFi RSSI level and time. The model can simply, but reliably estimate the impact of WiFi signal strength on phone energy in real time. We can set the formula of (${0.009|RSSI|}^{2}-0.7\left|RSSI\right|$) in (8) to zero and then get the value of |RSSI| with 78. This reveals that the phone energy consumption increases rapidly when the RSSI level is below −78 dBm, which is consistent with the conclusion in [8]. Furthermore, Equation (8) can be transformed to:
  (9) $$E\left(RSSI,t\right)=[0.009(\left|RSSI{\left|-38\right)}^{2}+1.26\right]t+1.76$$
  Formula (9) illustrates that under normal WiFi signals, for RSSI levels that range from −78 dBm to −38 dBm, the energy consumption decreases with the increase of WiFi signal strength.

## 5. Impact of WiFi Protocol Packets

In this section, we perform experiments to quantitatively study the impacts of the type and the total number of WiFi protocol packets on phone energy, and the packets are sent by WiFi APs to maintain the basic network communications of phones. The protocol types of the packets include UDP packets, TCP packets, ICMP packets and IGMP packets. The PCA (Principal Component Analysis) method is employed to find the most important protocol packet type that impacts phone energy. In other words, this section aims at answering research questions Q3 and Q4.

### 5.1. Energy Measurement

In this experiment, we use an AP setup on a PC to send protocol packets. The measured phone is also a Samsung Galaxy GT-S7898 running on Android 4.1.2. We keep the phone in the basic state so that all AP-phone communications during the experiment are initiated by the AP. We keep the AP-phone distance as 1 m so that the WiFi signal strength’s impact on phone energy is stable during the experiment.

We utilize Anysend [38], a software tool running on the AP’s PC, to send packets. All the packets’ sizes are fixed, namely 64 bytes. The packets sent are classified into four types, namely UDP packets, TCP packets, ICMP packets and IGMP packets, as shown at Column 1 in Table 3. For each package type, four groups of packets are sent, and each group contains 5, 10, 15 and 20 packets, respectively, as depicted in Row 2 in Table 3. Thus, 16 groups of packets are sent by the Anysend tool, and the packets in one group have the same protocol type.

We run the experiments 10 times. At each run, for each group of packets sent by the AP, we compute the energy consumed by the phone to handle all those packets, as follows:

(1) Measure energy consumption without and with packet-sending: We measure the phone’s energy consumption with and without packet-sending, respectively, where both measurements last for the same time period, e.g., 30 s. During the first measurement, the software Anysend sends no packets. During the second measurement, the software Anysend sends the group of packets. By using the method depicted in Section 3, we get the phone energy consumption $E_{0}$ and $E_{1}$ in the two measurements, respectively.
(2) Compute energy consumption Edue to handling packets: The time segment used for the phone to handle all the packets in the group is also that for the power meter to have an obvious current fluctuation. Thus, by observing current fluctuation on the power meter, we can discover the time segment for the phone to handle those packets. We find that the mentioned time segment is much less than our measurement time period (i.e., 30 s) and completely contained in the time period. Thus, by subtracting the phone energy consumption in 30 min without the AP’s packet-sending, namely $E_{0}$, from that with the AP’s packet-sending, namely $E_{1}$, we can compute the energy draw Edue to handling the group of packets, as the following formula shows:
  (10) $$E=E_{1}-E_{0}$$

We measure the phone energy draw for handling each group of packets 10 times and utilize the average value as the final energy consumption used for energy modeling. The mean values and the square error of the phone energy draw for each group of packets are listed in Table 3. To visualize the results in Table 3, Figure 6 plots the energy consumption (J) with the packet number under four types of packet types. Four types of scatterplots are made by different point types and point colors. For example, for the TCP packet type, the scatterplot consists of four black and round sampling points.

We can make the following observation from the graph in Figure 6: In a WiFi network,

- UDP packets initiated by an AP have little effect on the phone energy.
- TCP packets, ICMP packets and IGMP packets initiated by an AP have obvious effects on the phone energy. For each given packet type, the impacts on energy grow linearly with the total number of packets.
- The impacts of ICMP packets or IGMP packets on energy are higher than those of TCP packets.

### 5.2. Energy Model

**Figure 7:**
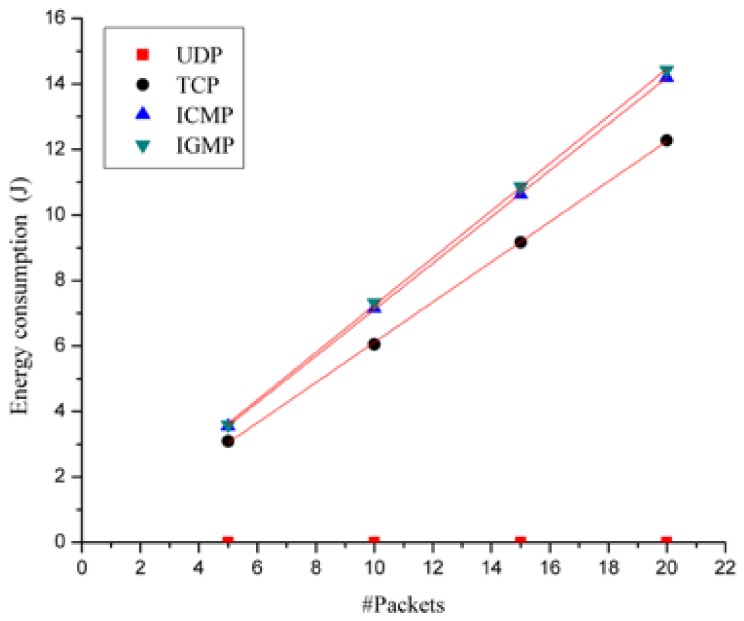
Energy consumed vs. packet amount.

For each given packet type, Figure 7 illustrates a polynomial fitted curve of the energy consumption with the packet number, and the corresponding linear regression equation (namely the energy model) is listed in Column 2 of Table 4. To validate the energy models, we perform three GoF tests. The test results are listed in the last three columns in Table 4, where both SSE and RMSE values are close to zero and R-square values are close to one. Thus, the results demonstrate that our models can simply but reliably estimate the impact of WiFi packet types and numbers.

In Column 2 of Table 4, the phone’s energy consumption is modeled as a constant of zero. This means that UDP packets sent by the WiFi APs have little impact on the phone’s energy consumption. This is because UDP packets sending from WiFi APs do not lead to the phone’s packet-sending actions, while the phone’s packet-receiving actions result only in very little energy consumption. The phone’s energy consumption models with TCP packets, ICMP packets and IGMP packets sent by the WiFi APs are listed in the last three rows of Column 2 of Table 4, respectively. The three types of packets have linear impacts on the phone’s energy draw with the packet amount. As the coefficients in the three equations show, ICMP packets and IGMP packets have a higher impact than TCP packets on the phone’s energy consumption.

Even if a phone does not initiate any communication with an AP in its WiFi network, the AP still initiates communication with the phone at times to perform such operations as arousing the phone. A private WiFi network initiates such communication chiefly by transmitting UDP packets and TCP packets, while a public WiFi network does so by sending not only UDP packets and TCP packets, but also multicast protocol packets such as ICMP packets and IGMP packets. As the models in Column 2 show, UDP packets have little impact on phone energy, while ICMP packets and IGMP packets have a higher linear impact than TCP packets. Thus, the energy of a smart phone in the basic state dissipates faster in a public WiFi network than in a private one. The results of this study are consistent with many phone user’s experience in public and private WiFi networks.

## 6. Discussion

When investigating the impact of WiFi signals on phone energy, we focus on good signals, whose RSSIs are over −50 dBm, rather than weak signals. We observed that the phone energy draw goes up with the decline of the signal strength under good signal conditions. The increment of energy drain could be caused by the rate adaptation at the physical layer with the changed signal strength, even if neither data re-transmission nor re-association with the AP is triggered by the good signals.

We create the energy model with the WiFi signal strength measured in RSSI as the parameter in Section 4. The WiFi signal strength can indicate the quality of the downlink between a user’s phone and its WiFi AP, to a large extent. Because the uplink and downlink between a user’s phone and a WiFi AP share the same communication channel, the quality of the downlink (or WiFi signal strength) also implies that of the uplink. For this reason, although the phone’s energy draw directly depends on the quality of the uplink, it is also reasonable to model the energy draw with WiFi signal strength as the parameter.

In order to corroborate the experience of phone users that phone energy drains faster under higher WiFi strength than under a lower one, we construct the energy model with the WiFi signal strength as the parameter. However, in order to reveal the root causes of the phone energy draw, in-depth studies need be done with the focus on such parameters as retransmission rate, error rate, transmission power, and so on.

For the impacts of WiFi network type (namely public or private network) on phone energy drain, we focus on the impacts caused by the different types of packets set by the WiFi APs. However, the impacts caused by other factors in different types of WiFi networks are not studied in this paper. Such factors include the quality of the communication channel, which might be influenced by the number of users, the phone’s mobility, and so on.

## 7. Conclusions and Future Work

In this paper, we investigated the impacts of normal WiFi environments on the energy consumption of mobile phones by detailed measurement. Experiments were conducted on the phone in the basic state at night to minimize interferences. We first empirically studied the impact of good signal strength on the drawn phone energy and created a signal strength-aware model based on measurement time to depict the impact, by comparing three regression methods, namely linear regression, quadratic linear regression and logarithmic linear regression. We then focused on four protocol types of packets initiated by the AP, where all four packets are basic packets in private or public WiFi networks. The protocol types include UDP packets, TCP packets, ICMP packets and IGMP packets. For each packet type, we conducted experiments with the fixed packet size of 64 bytes to analyze the relationship between the phone energy drain and the packet amount and finally proposed a packet-driven energy model. Our research results confirm the following experience of many phone users: higher signal strength implies lower energy drain even under normal WiFi signal conditions; phone energy dissipates faster in public WiFi networks than in private ones. The modeling method proposed in this paper enables developers to analyze the phone energy draw in WiFi environments conveniently, because the method only requires inexpensive power meter as the measurement tool and the model parameters are easily available.

Although the models proposed in this paper is phone-type dependent, the method to create the models could be applied to any phones. In order to improve our model methods, we plan to collect more experimental data on more types of phones. We also plan to study the impacts of other WiFi environmental factors on the phone energy, and then create energy models with the most prominent influence factors.

The models created in this paper primarily aim to help non-network specialists understand the impact of phone energy consumption on user experience in WiFi networks. Thus, we create the models based on easy-to-understand variables for non-network specialists, such as the WiFi signal strength and the count of the packets sent by the WiFi APs. However, the phone energy consumption can be directly affected by the following factors: the phone’s transmission parameters (e.g., transmission power retransmission rate, error rate, etc.), the packets sent and received by the phone, etc. By considering those direct factors in the future, the energy models might reveal the root causes of the phone energy consumption. As another future work, by considering such factors as the phone’s mobility and the number of users in the WiFi network, the energy model could better explain the different phone energy consumption between a public network and a private one. 

## Figures and Tables

**Table 1:** Distance, RSSI and energy consumption in 30 s.

Distance (m)	RSSI (dBm)	Energy Consumption (J)
0.5	−38	36 ± 0.44
1	−40	40 ± 0.22
1.5	−42	44 ± 0.22
2	−44	48 ± 0.67
2.5	−46	51 ± 0.22
3	−47	55 ± 0.44
3.5	−48	62 ± 0.44
4	−49	70 ± 0.22

**Table 2:** RSSI, energy model and GoF test results.

RSSI	Energy Model	SSE	R-Square	RMSE
−38 dBm	$E_{1}\left(t\right)=1.173t+1.76$	0.56	0.99	0.21
−40 dBm	$E_{2}\left(t\right)=1.311t+1.76$	8.78	0.98	0.56
−42 dBm	$E_{3}\left(t\right)=1.421t+1.76$	8.36	0.99	0.52
−44 dBm	$E_{4}\left(t\right)=1.532t+1.76$	5.54	0.99	0.30
−46 dBm	$E_{5}\left(t\right)=1.629t+1.76$	8.75	0.98	0.55
−47 dBm	$E_{6}\left(t\right)=1.735t+1.76$	8.86	0.98	0.58
−48 dBm	$E_{7}\left(t\right)=1.995t+1.76$	6.75	0.99	0.42
−49 dBm	$E_{8}\left(t\right)=2.291t+1.76$	6.94	0.99	0.48

**Table 3:** Energy consumption, packet type and amount.

Packet Type	Energy Consumption (J)			
	5 Packets	10 Packets	15 Packets	20 Packets
UDP	0	0	0	0
TDP	3.09 ± 0.0012	6.05 ± 0.0034	9.17 ± 0.0026	12.28 ± 0.0008
ICMP	3.55 ± 0.0008	7.15 ± 0.0016	10.63 ± 0.0026	14.20 ± 0.0015
IGMP	3.59 ± 0.0012	7.31 ± 0.0027	10.86 ± 0.0029	14.43 ± 0.0031

**Table 4:** Energy model, packet type and amount.

Packet Type	Energy Model	SSE	R-Square	RMSE
UDP	E=0	N/A	N/A	N/A
TCP	E=0.612*#TCP_pkts	0.01	0.98	0.05
ICMP	E=0.71*#ICMP_pkts	0.01	0.99	0.03
IGMP	E=0.723*#IGMP_pkts	0.01	0.98	0.05
